# Data on the effects of ECM rigidity on actomyosin contractility and invadopodia activity in individual versus pairs of head and neck squamous cell carcinoma cells

**DOI:** 10.1016/j.dib.2021.107684

**Published:** 2021-12-06

**Authors:** Rachel Jerrell, Mitchell Leih, Aron Parekh

**Affiliations:** a Department of Otolaryngology, Vanderbilt University Medical Center, 522 Preston Research Building, 2220 Pierce Avenue, Nashville, TN 37232, USA; b Vanderbilt-Ingram Cancer Center, Vanderbilt University Medical Center, USA; c Department of Biomedical Engineering, Vanderbilt University, USA

**Keywords:** Cancer, Invasion, Actomyosin contractility, Invadopodia, Rigidity

## Abstract

Migration through the extracellular matrix (ECM) is essential for cancer cells to escape the primary tumor and invade neighboring tissues with the potential for metastasis [1]. To penetrate tissue barriers, migrating cancer cells degrade the ECM with actin-rich membrane protrusions called invadopodia [2]. We have previously found that invadopodial ECM degradation is regulated by ECM rigidity in a process mediated by contractile forces in individual head and neck squamous cell carcinoma (HNSCC) cells [3], [4]. However, cancer cells often migrate together and interact with each other to alter their actomyosin contractility in response to the biomechanical properties of the ECM [5]. Therefore, we tested whether ECM rigidity promotes biomechanical interactions between cancer cells to enhance proteolytic activity. Using a minimal model of two HNSCC cells in physical contact, we provide data here that actomyosin contractility, invadopodia formation, and ECM degradation increase in response to ECM rigidity when cells are in pairs versus individual cells using traction force and invadopodia assays.

## Specifications Table

**Table:** 

Subject	Cancer Research
Specific subject area	Mechanobiological cancer cell invasion.
Type of data	Tables Figures
How data were acquired	Traction force microscopy and quantitative immunofluorescence of invadopodia assays were performed using a Nikon Ti-E inverted microsope with 40 × 0.75 NA Plan Fluor and 40 × Plan Fluor oil immersion objectives, respectively.
Data format	Raw Analyzed
Parameters for data collection	The HNSCC cell line SCC-61 was used in 3–6 replicates plated overnight on fibronectin-conjugated polyacrylamide gels (PAAs) with increasing mechanical properties.
Description of data collection	Images of cells were captured using a Zyla 4.2 PLUS CMOS camera and Nikon Elements software.
Data source location	Institution: Vanderbilt University Medical Center City/Town/Region: Nashville, Tennessee Country: United States of America Latitude and longitude (and GPS coordinates, if possible) for collected samples/data: 36.141739, -86.802132
Data accessibility	With the article and as follows: Repository name: Mendeley Data Data identification number: DOI:10.17632/72377p68n2.1 Direct URL to data: https://data.mendeley.com/datasets/72377p68n2/1

## Value of the Data

• The data presented here reveal the impact of ECM rigidity on the contractile and invasive properties of HNSCC cells in contact with each other.
• The data may be of interest to researchers studying cancer biology and mechanisms of invasion including the roles of intercellular biophysical interactions between cancer cells migrating in multicellular and collective groups.
• The data may provide the basis for future studies to uncover force-dependent mechanisms that augment invadopodia activity in cohesive groups of cancer cells during proteolytic invasion.

## 1 Data Description

**Fig. 1:**
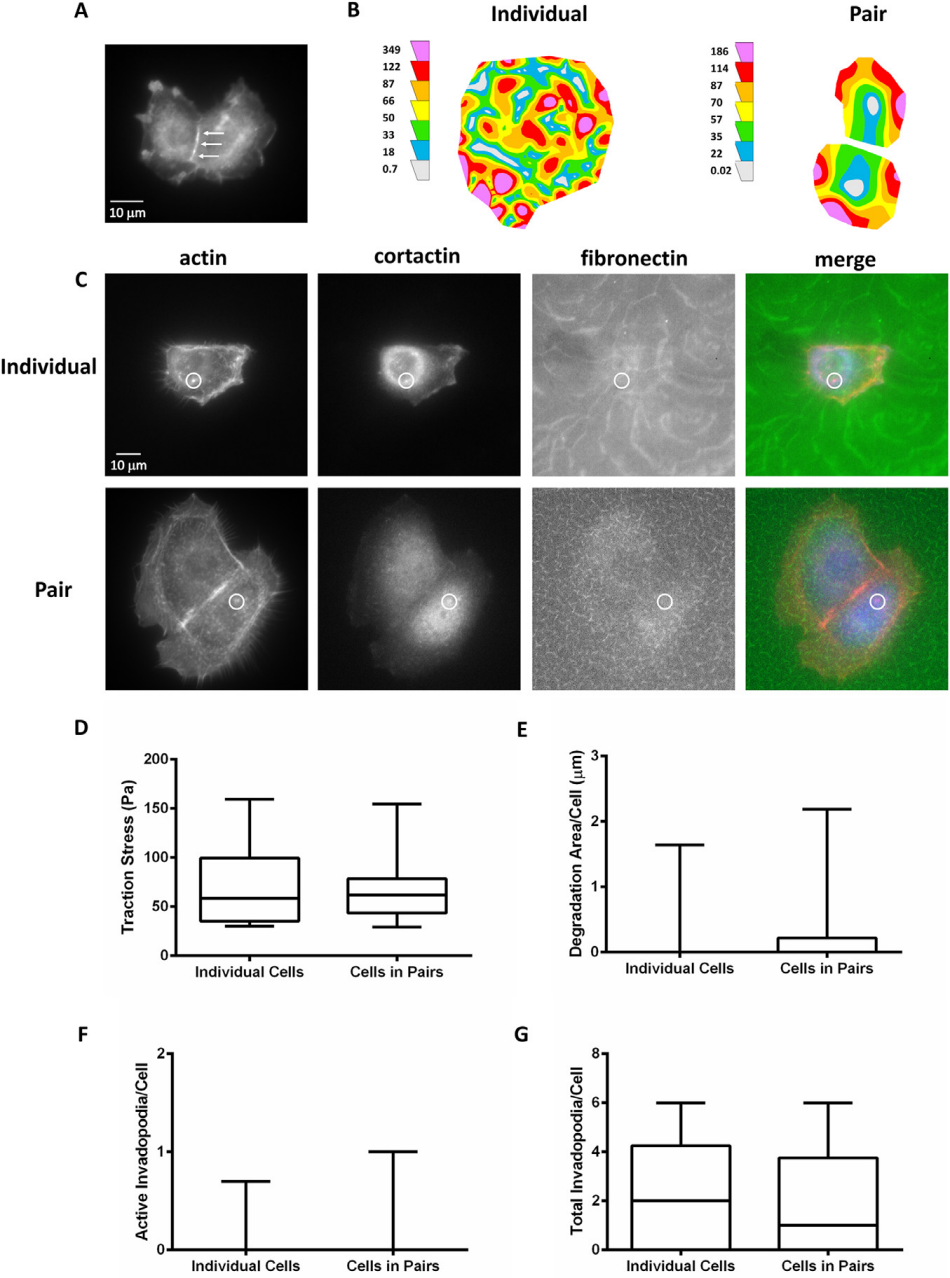
Traction forces and invadopodia activity do not change in pairs versus individual SCC-61 cells on soft polyacrylamide gels (PAAs). (A) Representative wide-field fluorescent image of p120-catenin as a marker for adherens junctions in a cell pair to verify physical contact and intercellular interactions. (B) Representative traction maps with colors representing local traction stress levels in an individual and pair of cells. (C) Representative wide-field fluorescent images of an individual and pair of cells in an invadopodia assay in which nascent or immature invadopodia were identified by the colocalization of actin and cortactin over non-degraded FITC-fibronectin (white circles). Quantitation of (D) traction stress, (E) ECM degradation area per cell, (F) number of mature or actively degrading invadopodia per cell (i.e., colocalized with ECM degradation), and (G) number of total invadopodia per cell (i.e., immature and mature). Data are presented as box and whisker plots with black lines indicating the medians, whiskers representing the 10th and 90th percentiles, and * indicating *p* < 0.05 for *n* = 32 individual and 43 pairs of cells from 4 independent traction force experiments and *n* = 42 individual and 26 pairs of cells for 3 independent invadopodia experiments.

**Fig. 2:**
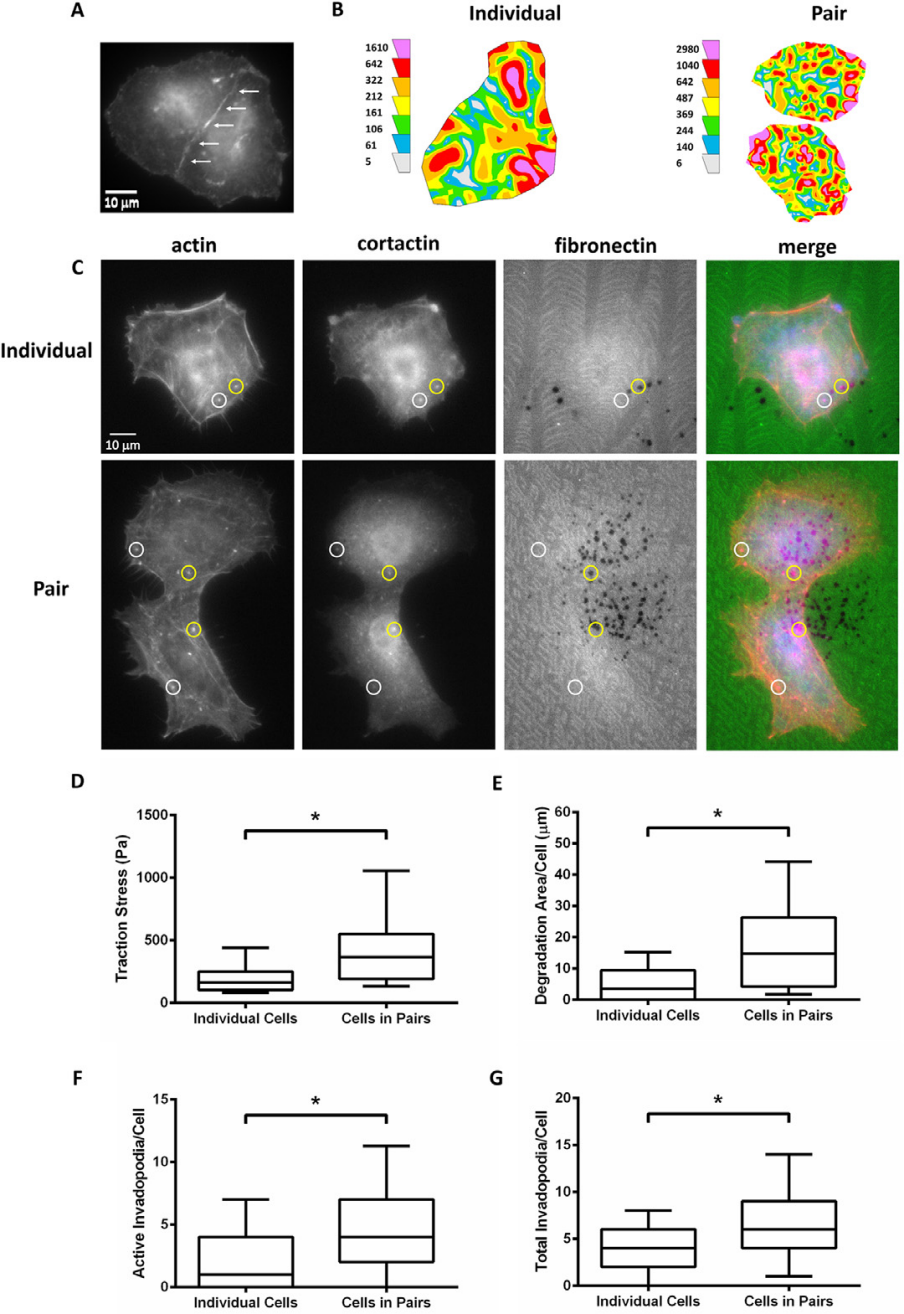
Traction forces and invadopodia activity increase in pairs versus individual SCC-61 cells on hard polyacrylamide gels (PAAs). (A) Representative wide-field fluorescent image of p120-catenin as a marker for adherens junctions in a cell pair to verify physical contact and intercellular interactions. (B) Representative traction maps with colors representing local traction stress levels in an individual and pair of cells. (C) Representative wide-field fluorescent images of an individual and pair of cells in an invadopodia assay in which nascent or immature invadopodia were identified by the colocalization of actin and cortactin over non-degraded FITC-fibronectin (white circles) while actively degrading or mature invadopodia were also colocalized with ECM degradation (i.e., black areas lacking FITC signal; yellow circles). Quantitation of (D) traction stress, (E) ECM degradation area per cell, (F) number of mature or actively degrading invadopodia per cell (i.e., colocalized with ECM degradation), and (G) number of total invadopodia per cell (i.e., immature and mature). Data are presented as box and whisker plots with black lines indicating the medians, whiskers representing the 10th and 90th percentiles, and * indicating *p* < 0.05 for *n* = 32 individual and 42 pairs of cells from 6 independent traction force experiments and *n* = 58 individual and 38 pairs of cells for 4 independent invadopodia experiments.

The analyzed and raw data provided here demonstrate the effects of ECM rigidity on the generation of traction forces and formation of invadopodia and their associated ECM degradation in individual HNSCC cells versus pairs of physically interacting HNSCC cells using the SCC-61 cell line. ECM rigidity was varied from soft (Fig. 1) to hard (Fig. 2) PAAs to mimic normal tissue and tumor-associated mechanical properties, respectively, *in vitro* [6], [7], [8]. Physical contact and interactions between HNSCC cells in pairs on the soft (Fig. 1A) and hard (Fig. 2A) PAAs were confirmed with immunofluorescence. On soft PAAs, traction forces (Fig. 1B & D; Table 1), ECM degradation, number of actively degrading or mature invadopodia, and number of total invadopodia (i.e., mature and immature or nascent invadopodia) (Fig. 1C & E–G; Table 2) did not change when comparing individual versus pairs of HNSCC cells. However, traction forces (Fig. 2B & D; Table 3), ECM degradation, number of active invadopodia, and number of total invadopodia (Fig. 2C & E–G; Table 4) increased for HNSCC cells within pairs when compared to individual cells on hard PAAs.

**Table 1:** Raw data from the traction force analyses of individual and pairs of SCC-61 cells on soft PAAs.

			Traction Stress
Experiment	Condition	Replicate	(Pa)
1	Individual	1	878
1	Individual	1	39.2
1	Individual	1	63.9
1	Individual	1	34.6
2	Individual	1	62.32
2	Individual	1	45.69
2	Individual	1	16.77
2	Individual	2	68.58
2	Individual	2	32.43
2	Individual	2	67.57
2	Individual	3	39.12
2	Individual	3	32.13
2	Individual	3	70.36
3	Individual	1	54.41
3	Individual	1	167.2
3	Individual	1	35.59
3	Individual	1	104.3
3	Individual	2	515
3	Individual	2	103
3	Individual	2	141.8
3	Individual	2	121.7
3	Individual	3	79.89
3	Individual	3	29.27
3	Individual	3	34.98
3	Individual	3	52.04
4	Individual	1	89.57
4	Individual	1	126.1
4	Individual	1	41.83
4	Individual	2	66.64
4	Individual	2	44.44
4	Individual	3	21.21
4	Individual	3	33.63
1	Pair	1	24.1
1	Pair	1	31.1
1	Pair	1	66.8
1	Pair	1	66.1
1	Pair	1	56.1
1	Pair	1	57.7
1	Pair	1	39.9
1	Pair	1	23.2
2	Pair	1	28.77
2	Pair	1	43.63
2	Pair	1	68.71
2	Pair	1	51.08
2	Pair	1	78.81
2	Pair	1	96.05
2	Pair	1	38.22
2	Pair	1	39.51
2	Pair	1	50.58
2	Pair	1	78.82
2	Pair	2	75.47
2	Pair	2	29.25
2	Pair	2	78.44
2	Pair	2	85.59
2	Pair	3	224
2	Pair	3	432
2	Pair	3	69.32
2	Pair	3	53.79
2	Pair	3	53.35
2	Pair	3	62
2	Pair	3	40.18
2	Pair	3	56.73
3	Pair	1	78.08
3	Pair	1	27.79
3	Pair	1	29.08
3	Pair	1	51.51
3	Pair	1	101
3	Pair	1	108
3	Pair	1	56.59
3	Pair	1	74.5
3	Pair	2	56.89
3	Pair	2	62.24
3	Pair	2	144.2
3	Pair	2	179
3	Pair	2	63.94
3	Pair	2	224.3
3	Pair	2	454.8
3	Pair	2	580.3
3	Pair	2	92.09
3	Pair	2	79.83
3	Pair	3	61.68
3	Pair	3	70.91
3	Pair	3	28.28
3	Pair	3	30.07
3	Pair	3	111.4
3	Pair	3	203.8
3	Pair	3	72.87
3	Pair	3	73.16
4	Pair	1	44.38
4	Pair	1	58.3
4	Pair	1	62.58
4	Pair	1	35.51
4	Pair	1	54.41
4	Pair	1	59.18
4	Pair	1	42.13
4	Pair	1	36.29
4	Pair	1	43.39
4	Pair	1	77.96
4	Pair	2	389.5
4	Pair	2	97.56
4	Pair	2	47.43
4	Pair	2	74.2
4	Pair	2	25.87
4	Pair	2	46.81
4	Pair	2	67.69
4	Pair	2	83.57
4	Pair	2	53.87
4	Pair	2	47.46
4	Pair	3	61.81
4	Pair	3	63.39
4	Pair	3	61.86
4	Pair	3	49.95
4	Pair	3	67.34
4	Pair	3	87.9
4	Pair	3	25.83
4	Pair	3	59.92
4	Pair	3	43.22
4	Pair	3	30.29

**Table 2:** Raw data from the immunofluorescence analyses of individual and pairs of SCC-61 cells on soft PAAs in invadopodia assays.

			Degradation	Active	Total
Experiment	Condition	Replicate	(μm^2^)	Invadopodia	Invadopodia
1	Individual	1	0.00	0	0
1	Individual	1	0.00	0	1
1	Individual	1	0.00	0	0
1	Individual	1	15.04	3	6
1	Individual	1	0.00	0	3
1	Individual	1	0.00	0	6
1	Individual	1	0.00	0	5
1	Individual	1	0.00	0	9
1	Individual	1	0.00	0	0
1	Individual	2	0.00	0	2
1	Individual	2	0.00	0	0
1	Individual	2	0.00	0	0
1	Individual	2	0.00	0	4
1	Individual	2	0.00	0	2
1	Individual	2	0.00	0	0
1	Individual	3	0.00	0	0
1	Individual	3	0.00	0	1
1	Individual	3	0.00	0	0
1	Individual	3	1.83	0	2
1	Individual	3	0.00	0	1
1	Individual	3	0.00	0	1
1	Individual	3	0.00	0	0
1	Individual	3	0.00	0	0
2	Individual	1	1.20	2	8
2	Individual	1	0.00	0	8
2	Individual	1	0.00	0	3
2	Individual	1	0.00	0	5
2	Individual	1	0.00	0	6
2	Individual	1	4.54	1	3
2	Individual	2	6.16	1	4
2	Individual	2	0.00	0	6
2	Individual	2	0.00	0	4
2	Individual	2	0.24	0	4
2	Individual	2	0.00	0	2
3	Individual	1	0.00	0	0
3	Individual	1	0.00	0	1
3	Individual	1	0.00	0	5
3	Individual	1	0.00	0	0
3	Individual	1	1.17	0	0
3	Individual	2	0.48	0	2
3	Individual	2	0.00	0	4
3	Individual	2	0.00	0	0
1	Pair	1	0.00	0	0
1	Pair	1	0.00	0	0
1	Pair	1	2.44	3	6
1	Pair	1	0.00	0	0
1	Pair	1	0.00	0	0
1	Pair	1	0.00	0	4
1	Pair	1	0.00	0	0
1	Pair	1	7.28	2	4
1	Pair	1	1.59	0	0
1	Pair	1	0.00	0	0
1	Pair	2	0.00	0	3
1	Pair	2	26.52	6	6
1	Pair	2	12.67	4	4
1	Pair	2	0.00	0	0
1	Pair	2	0.00	0	5
1	Pair	2	0.00	0	0
1	Pair	2	0.00	0	0
1	Pair	2	0.00	0	0
1	Pair	2	0.00	0	3
1	Pair	2	0.00	0	1
1	Pair	2	0.00	0	3
1	Pair	2	0.00	0	1
1	Pair	3	0.00	0	2
1	Pair	3	0.00	0	1
1	Pair	3	0.00	0	0
1	Pair	3	0.00	0	5
2	Pair	1	0.00	0	7
2	Pair	1	0.29	0	4
2	Pair	2	0.00	0	0
2	Pair	2	0.00	0	0
2	Pair	3	0.00	0	3
2	Pair	3	0.00	0	8
2	Pair	3	0.32	0	0
2	Pair	3	1.51	0	0
2	Pair	3	0.32	1	3
2	Pair	3	0.40	1	3
2	Pair	3	0.00	0	0
2	Pair	3	0.00	0	6
2	Pair	3	0.00	0	3
2	Pair	3	0.00	0	8
3	Pair	1	0.00	0	2
3	Pair	1	0.00	0	8
3	Pair	1	0.00	0	1
3	Pair	1	0.69	0	0
3	Pair	1	6.30	0	0
3	Pair	1	0.00	0	0
3	Pair	1	0.00	0	0
3	Pair	1	0.00	0	0
3	Pair	2	0.00	0	0
3	Pair	2	0.00	0	2
3	Pair	2	0.00	0	2
3	Pair	2	1.14	0	3

**Table 3:** Raw data from the traction force analyses of individual and pairs of SCC-61 cells on hard PAAs.

			Traction Stress
Experiment	Condition	Replicate	(Pa)
1	Individual	1	210.7
1	Individual	1	1632
1	Individual	2	250
1	Individual	2	233.5
2	Individual	1	95.55
2	Individual	1	79.88
2	Individual	1	74.52
2	Individual	1	114.5
3	Individual	1	176.1
3	Individual	1	217.5
3	Individual	1	250.1
3	Individual	2	119.5
3	Individual	2	394.5
3	Individual	2	459.7
3	Individual	2	251.9
4	Individual	1	84.91
4	Individual	1	117.4
4	Individual	1	134.9
4	Individual	1	335.6
4	Individual	1	100.8
4	Individual	2	111.9
4	Individual	2	81.19
4	Individual	2	93.51
4	Individual	2	183.2
4	Individual	2	345.1
5	Individual	1	81.51
5	Individual	2	272
6	Individual	1	185.1
6	Individual	1	144.4
6	Individual	1	151.7
6	Individual	1	110.5
6	Individual	2	804.5
1	Pair	1	24.1
1	Pair	1	31.1
1	Pair	1	66.8
1	Pair	1	66.1
1	Pair	1	56.1
1	Pair	1	57.7
1	Pair	2	39.9
1	Pair	2	23.2
1	Pair	2	28.77
1	Pair	2	43.63
1	Pair	2	68.71
1	Pair	2	51.08
2	Pair	1	78.81
2	Pair	1	96.05
2	Pair	1	38.22
2	Pair	1	39.51
2	Pair	1	50.58
2	Pair	1	78.82
2	Pair	1	75.47
2	Pair	1	29.25
3	Pair	1	78.44
3	Pair	1	85.59
3	Pair	1	224
3	Pair	1	432
3	Pair	1	69.32
3	Pair	1	53.79
3	Pair	1	53.35
3	Pair	1	62
3	Pair	1	40.18
3	Pair	1	56.73
3	Pair	2	78.08
3	Pair	2	27.79
3	Pair	2	29.08
3	Pair	2	51.51
3	Pair	2	101
3	Pair	2	108
3	Pair	2	56.59
3	Pair	2	74.5
4	Pair	1	56.89
4	Pair	1	62.24
4	Pair	1	144.2
4	Pair	1	179
4	Pair	1	63.94
4	Pair	1	224.3
4	Pair	1	454.8
4	Pair	1	580.3
4	Pair	1	92.09
4	Pair	1	79.83
4	Pair	2	61.68
4	Pair	2	70.91
4	Pair	2	28.28
4	Pair	2	30.07
4	Pair	2	111.4
4	Pair	2	203.8
4	Pair	2	72.87
4	Pair	2	73.16
4	Pair	2	44.38
4	Pair	2	58.3
5	Pair	1	62.58
5	Pair	1	35.51
5	Pair	1	54.41
5	Pair	1	59.18
5	Pair	1	42.13
5	Pair	1	36.29
5	Pair	2	43.39
5	Pair	2	77.96
5	Pair	1	389.5
6	Pair	1	97.56
6	Pair	1	47.43
6	Pair	1	74.2
6	Pair	1	25.87
6	Pair	1	46.81
6	Pair	1	67.69
6	Pair	1	83.57
6	Pair	1	53.87
6	Pair	1	47.46
6	Pair	2	61.81
6	Pair	2	63.39
6	Pair	2	61.86
6	Pair	2	49.95
6	Pair	2	67.34
6	Pair	2	87.9
6	Pair	2	25.83
6	Pair	2	59.92

**Table 4:** Raw data from the immunofluorescence analyses of individual and pairs of SCC-61 cells on hard PAAs in invadopodia assays.

			Degradation	Active	Total
Experiment	Condition	Replicate	(μm^2^)	Invadopodia	Invadopodia
1	Individual	1	34.33	0	4
1	Individual	1	15.22	4	4
1	Individual	1	4.70	0	0
1	Individual	1	0.00	0	2
1	Individual	1	0.00	0	0
1	Individual	1	0.00	0	6
1	Individual	1	0.00	0	3
1	Individual	1	0.00	0	4
1	Individual	2	0.00	0	0
1	Individual	2	0.00	0	2
1	Individual	2	0.00	0	8
1	Individual	2	0.00	0	0
1	Individual	3	3.72	0	0
1	Individual	3	6.00	7	9
1	Individual	3	12.14	7	8
1	Individual	3	16.10	2	2
1	Individual	3	0.00	0	2
1	Individual	3	1.94	2	6
2	Individual	1	6.22	6	8
2	Individual	1	3.40	5	8
2	Individual	1	0.00	0	5
2	Individual	1	1.65	1	7
2	Individual	1	1.67	2	11
2	Individual	2	5.69	1	4
2	Individual	2	13.76	10	14
2	Individual	2	0.00	0	5
2	Individual	2	0.58	0	4
2	Individual	2	0.00	0	4
2	Individual	2	0.00	0	2
2	Individual	3	1.43	4	4
2	Individual	3	0.00	0	5
2	Individual	3	0.00	0	4
2	Individual	3	6.48	4	6
2	Individual	3	3.93	1	2
3	Individual	1	0.00	4	4
3	Individual	1	2.58	0	2
3	Individual	1	17.64	4	4
3	Individual	1	12.75	4	6
3	Individual	1	15.84	7	7
3	Individual	1	12.06	7	8
3	Individual	1	9.33	6	6
3	Individual	2	10.76	5	5
3	Individual	2	11.16	2	5
3	Individual	2	14.85	7	9
3	Individual	3	0.00	0	1
3	Individual	3	4.92	2	5
3	Individual	3	3.80	2	2
3	Individual	3	9.64	4	8
4	Individual	1	2.92	0	3
4	Individual	1	6.72	1	3
4	Individual	1	6.03	4	4
4	Individual	1	15.84	5	5
4	Individual	1	0.00	0	0
4	Individual	1	4.73	0	1
4	Individual	1	8.69	8	8
4	Individual	2	9.06	0	1
4	Individual	2	0.00	0	1
4	Individual	2	1.46	1	2
1	Pair	1	5.05	2	3
1	Pair	1	6.75	0	3
1	Pair	1	22.13	6	6
1	Pair	1	0.00	0	3
1	Pair	1	9.06	4	6
1	Pair	1	12.75	1	1
1	Pair	1	26.33	5	10
1	Pair	1	3.48	3	6
1	Pair	1	4.07	4	4
1	Pair	1	0.00	0	2
1	Pair	1	2.05	0	1
1	Pair	1	1.91	4	6
1	Pair	1	61.83	10	14
1	Pair	1	31.19	0	1
1	Pair	2	85.90	8	11
1	Pair	2	30.45	7	8
1	Pair	2	22.77	7	8
1	Pair	2	30.39	9	11
1	Pair	2	1.41	0	0
1	Pair	2	15.04	5	6
1	Pair	2	1.81	0	1
1	Pair	2	4.14	0	1
1	Pair	3	3.11	0	1
1	Pair	3	1.70	1	1
1	Pair	3	1.38	7	8
1	Pair	3	8.58	0	1
1	Pair	3	18.39	2	4
1	Pair	3	12.99	2	4
1	Pair	3	44.87	15	22
1	Pair	3	3.43	3	3
1	Pair	3	22.58	1	3
1	Pair	3	3.72	6	7
2	Pair	1	23.35	7	7
2	Pair	1	30.47	3	5
2	Pair	1	21.81	13	14
2	Pair	1	26.22	4	4
2	Pair	1	28.99	7	7
2	Pair	1	3.85	0	4
2	Pair	1	50.85	18	18
2	Pair	1	63.07	10	12
2	Pair	1	9.56	4	8
2	Pair	1	4.17	4	5
2	Pair	1	28.40	2	4
2	Pair	1	43.81	1	1
2	Pair	2	30.08	29	29
2	Pair	2	24.04	5	6
2	Pair	2	12.25	7	9
2	Pair	2	0.77	0	6
2	Pair	2	0.43	1	9
2	Pair	2	14.83	8	12
2	Pair	2	5.45	10	10
2	Pair	2	3.11	3	6
2	Pair	3	36.93	11	11
2	Pair	3	8.48	7	7
2	Pair	3	14.19	5	5
2	Pair	3	14.69	4	4
3	Pair	1	24.92	8	10
3	Pair	1	60.63	8	12
3	Pair	1	22.40	4	7
3	Pair	1	10.63	5	7
3	Pair	1	8.79	4	4
3	Pair	1	15.28	2	3
3	Pair	2	18.97	3	4
3	Pair	2	4.49	8	9
3	Pair	2	17.59	1	2
3	Pair	2	46.55	5	5
3	Pair	2	26.17	4	4
3	Pair	2	32.65	4	5
3	Pair	2	32.92	4	8
3	Pair	2	16.21	3	7
4	Pair	1	15.52	2	6
4	Pair	1	12.22	22	22
4	Pair	1	5.45	12	15
4	Pair	1	7.47	15	18
4	Pair	2	5.87	4	7
4	Pair	2	19.74	8	8

## 2 Experimental Design, Materials and Methods

### 2.1 Cell culture

The HNSCC cell line SCC-61 was cultured in Dulbecco's modified Eagle's medium (ThermoFisher) containing 20% fetal bovine serum (ThermoFisher) and 0.4 μg/ml hydrocortisone (MilliporeSigma) as previously described [3].

### 2.2 Soft and hard PAAs

Soft and hard PAAs were prepared from stocks of 40% acrylamide (Bio-Rad), 2% BIS-acrylamide (Bio-Rad), 10 mg/ml acrylic acid N-hydroxysuccinimide (MilliporeSigma), and 1 mg/ml fibronectin (ThermoFisher) at final concentrations of 8%/0.05%/0.1% with 200 μg/ml fibronectin and 8%/0.35%/0.1% with 215 μg/ml fibronectin, respectively, and cast on activated coverslips in 35 mm glass bottom dishes (MatTek) as previously described [3,9]. Soft and hard PAAs were previously measured with rheometry yielding elastic moduli of 1023 and 7307 Pa, respectively [3].

### 2.3 Traction force assays and force analyses

For traction force assays, 200 nm red fluorescent beads (ThermoFisher) were included at a final ratio of 1:125 in the PAAs to detect substrate displacements caused by cellular forces as previously described [3,9]. Briefly, images were taken of cells and then the underlying beads at the PAA surface before (“stressed” image) and after (“null” image) removal of the cells. Traction forces were calculated based on the optical flow method for bead tracking, the PAA mechanical properties, and the maximum likelihood method using the licensed software LIBTRC [3,4]. Traction forces calculated for pairs represent values for each cell within the pair and are reported for all cells as the mean of the magnitude of the surface traction stress vectors for each cell.

### 2.4 Invadopodia assays and immunofluorescence analyses

For invadopodia assays, PAAs were overlaid with 1% crosslinked gelatin (Polysciences) and centrifuged 50 μg/ml fluorescein isothiocyanate-labeled fibronectin to detect ECM degradation as previously described [3,4,9]. Briefly, invadopodia were identified by the colocalization of F-actin and cortactin using Alexa Fluor 546 phalloidin (1:750; ThermoFisher; catalog number: A22283) and 4F11 anti-cortactin mouse primary monoclonal antibody/Alexa Fluor 633 goat anti-mouse IgG secondary antibody (1:750; MilliporeSigma; catalog number: 05-180-I/1:500; ThermoFisher; catalog number: A-21050), respectively, and ECM degradation was quantitated by thresholding for the loss of FITC signal under each cell. Invadopodia data represent values for each cell within the pair. To confirm contact and physical interactions between cells in pairs, SCC-61 cells were immunostained with an anti-p120-catenin rabbit primary polyclonal antibody (generated and gifted by Al Reynolds, Vanderbilt University) and visualized with an Alexa Fluor 633 goat anti-rabbit IgG secondary antibody (ThermoFisher) as a marker for adherens junctions which facilitate intercellular transmission of contractile forces [10].

### 2.5 Statistics

As previously described, data were evaluated for normality using the Kolmogorov-Smirnov test, determined to be nonparametric, and compared using a Mann-Whitney test with a *p*-value < 0.05 considered statistically significant [3,4].

## Ethics Statement

N/A.

## CRediT authorship contribution statement

**Rachel Jerrell:** Investigation, Visualization. **Mitchell Leih:** Formal analysis. **Aron Parekh:** Formal analysis, Methodology, Visualization, Writing – original draft.

## Declaration of Competing Interest

The authors declare that they have no known competing financial interests or personal relationships which have or could be perceived to have influenced the work reported in this article.
